# Ngaramadhi Space: An Integrated, Multisector Model of Care for Students Experiencing Problematic Externalising Behaviour

**DOI:** 10.5334/ijic.7612

**Published:** 2023-12-14

**Authors:** Santuri Rungan, Susan Gardner, Huei-Ming Liu, Susan Woolfenden, Jennifer Smith-Merry, John Eastwood

**Affiliations:** 1 Sydney Local Health District, University of Sydney, Sydney Institute for Women, Children & their Families, AU; 2 Sydney Local Health District, AU; 3 The George Institute for Global Health, University of New South Wales, AU; 4 Sydney Local Health District, University of Sydney, AU; 5 Sydney Institute Women, Children and their Families, University of New South Wales, AU; 6 Centre for Disability Research and Policy, Sydney School of Health Sciences, The University of Sydney, AU; 7 University of New South Wales, Sydney, AU; 8 Ingham Institute of Applied Medical Research, Liverpool, NSW, AU; 9 University of Sydney, Sydney Institute for Women, Children and their Families and Sydney Local Health District, AU

**Keywords:** school based health care, health promoting schools, National Children’s Mental Health and Wellbeing Strategy, partnerships between health and education, Healthy Homes and Neighbourhoods (HHAN)

## Abstract

**Introduction:**

Behavioural and emotional disorders are a significant cause of morbidity for young people aged 10–19 years. School-based health care (SBHC) provides an innovative approach to addressing these issues within Australia.

**Description:**

We describe an innovative and integrative SBHC model called Ngaramadhi Space (NS) based at a specialised behavioural school called Yudi Gunyi school (YGS) in metropolitan Sydney, Australia. NS was developed in partnership with the Aboriginal community to provide holistic, integrated, multidisciplinary child and family centred care to students experiencing problematic externalising behaviour. We contextualise the historical factors leading to the development of NS, highlighting the importance of effective partnerships between sectors, and providing the theoretical framework and key components underpinning the model of care.

**Discussion:**

In Australia, schools are an under-utilised resource for the delivery of health and support alongside education. Collaboration between sectors can be challenging but allows a more coordinated approach to the management of complex social and health issues. By forming effective partnerships with schools and communities, the health sector has an opportunity to improve access to health and social care in a culturally safe and acceptable way. This is in line with national and international frameworks for improving health service delivery and addressing inequity.

**Conclusion:**

The health sector can play a pivotal role in improving the wellbeing of children by forming effective partnerships with schools and communities. The NS model is a practice-based example of this.

## Introduction

Behavioural and emotional disorders are a significant cause of morbidity for young people aged between 10-19 years [1,2]. Progress in the health and wellbeing of this group has plateaued with mental health and substance abuse issues being significant issues [1,2,3]. In Australia, 14% of children experience mental health disorders such as attention-deficit and hyperactivity disorder (ADHD, 7.4%), anxiety disorders (6.9%), depression (2.8%), and conduct disorders (2.1%) [4]. Within Sydney Local Health District (SLHD) depression, anxiety, substance abuse, and behavioural disorders rank in the top five most prevalent diseases for young people with a 160% increase in emergency department (ED) presentations for suicide/self-injury and a 139% increase in total ED presentations for mental health issues between 2015–2021 [5].

The impact of mental health disorders for young people is significant and contributes to negative individual, social and economic outcomes [6,7,8,9]. In Australia, access to mental health services is limited, with only half of affected children receiving appropriate services and visible inequities between Aboriginal and non-Aboriginal children [4,10,11,12,13,14,15]. Aboriginal children experience poorer health and wellbeing outcomes, lower levels of service use and barriers to accessing services [16,17,18,19,20]. There is evidence to suggest a widening of these inequities as a result of the COVID 19 pandemic [21,22,23,24,25].

In Australia’s first ‘National Children’s Mental Health and Wellbeing Strategy (NCMHWS)’ a need to develop innovative child-centred approaches to mental health and equitable access to services was outlined [26]. School-based healthcare (SBHC) provides a solution to this issue [27,28,29,30]. Worldwide, most children are enrolled continuously in school [31,32]. This provides an opportunity to improve access to healthcare in an environment that is safe and acceptable to children and families [27,28,29,33].

SBHC is well established in the United States (US), United Kingdom (UK) and New Zealand (NZ) were they have been designed to meet the unique needs of local communities [34,35,36,37,38,39]. SBHC provides a range of physical health and mental health services and have been associated with increased access to care, improved health and education outcomes, high levels of student and parent satisfaction, and reduced health care costs for priority populations [28,40,41,42,43,44,45,46,47,48,49].

Within Australia, there is a growing interest in SBHC models of care with the ground-up development of initiatives such as ‘Our Mia Mia’ (OMM) and ‘Our Place’ [50,51,52,53]. In addition, the ‘Australasian School-Based Health Alliance (ASBHA)’ has been formed to bring together a knowledge base and a network of practitioners to advance this field [54].

In this paper, an innovative SBHC model called Ngaramadhi Space (NS) is described. NS is unique because it is based at a specialised secondary school, Yudi Gunyi school (YGS), for students experiencing problematic externalising behaviour [55]. Located in metropolitan Sydney, Australia, NS was developed in partnership with the Aboriginal community to provide holistic, integrated, multidisciplinary child and family-centred care. The NS model will be contextualised within the historical factors which led to its development, highlighting the importance of building effective partnerships between the health sector, the education sector and the community. In addition, the theoretical framework and key components underpinning the model of care will be outlined [27,56,57].

## Yudi Gunyi School (YGS) and Ngaramadhi Space (NS)

YGS is a ‘School for Special Purposes’ (SSP), catering for students aged 10-16 years experiencing problematic externalising behaviour in a mainstream school setting [55,58]. Up to 34 students are enrolled at any one time, with enrolments occurring throughout the year [55]. Through an individualised case-management approach, the school aims to successfully transition students back into a mainstream school or to the wider community if they have completed compulsory schooling [55].

**Figure 1:**
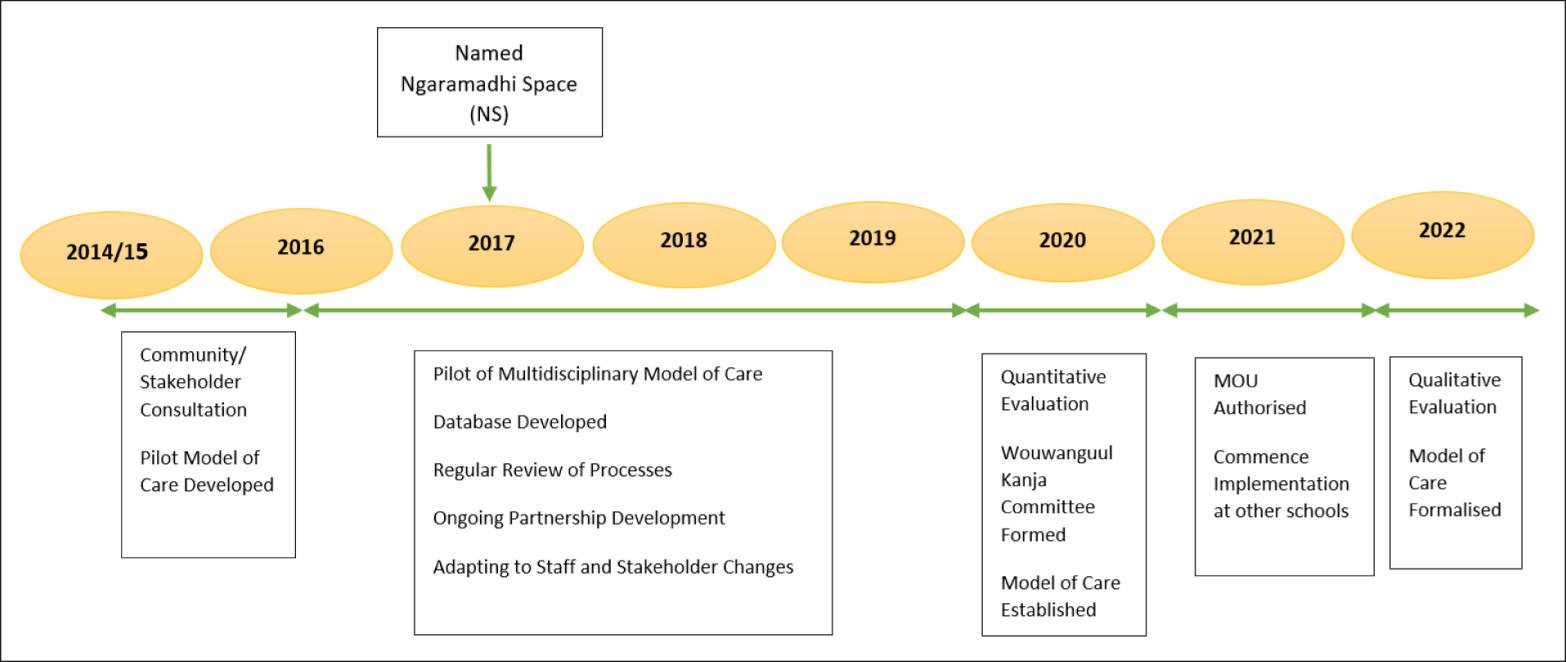
Timeline for Development of the Ngaramadhi Space Model of Care.

The NS model of care was developed over almost a decade and represents an integrated approach between the health, education and social care sectors [59,60,61]. The process of developing the model (visualised in Figure 1) began in 2014 as part of a broader community consultation initiative undertaken as part of the Healthy Homes and Neighbourhoods (HHAN) integrated care initiative [62,63]. The aim of the consultation process, which involved community organisations and service providers, was to strategically identify potential sites for co-location of services [62,63]. Out of this process, YGS was identified as a priority school. YGS was recognised as an endpoint for a confluence of social risk factors, such as low socioeconomic status, parental mental health issues, and intergenerational adversity [58]. As a result, discussions between key stakeholders across sectors were commenced. The voices of community members including the local Aboriginal community were an important part of this process and provided direction with developing the holistic model of care.

Between 2015 and 2016, the integrated model was developed and it was formally piloted between 2016 to 2019. The shared goal of the model was to address the physical health, mental health, educational, and social needs of students and their families. The multidisciplinary team included a paediatrician, youth health nurse, social worker, school counsellor, speech therapist, and occupational therapist. A child and adolescent psychiatrist was partnered with to provide consultative advice on students.

In 2017, the name ‘Ngaramadhi Space (NS)’ was gifted to the initiative by the Aboriginal community [55]. ‘Ngaramadhi Space (NS)’ refers to the multidisciplinary clinic as well as the rooms within the school where the clinics are held. These rooms were purpose-built to have multiple uses, including space for clinical assessments, counselling and therapy sessions, while being welcoming to young people and families.

During the pilot phase and based on positive feedback received from the community and partners, the NS model started becoming integrated within YGS. This included an evaluation study started in 2020, establishing a community reference group called the ‘Wouwanguul Kanja’ committee, forming a memorandum of understanding (MOU) that was authorised in 2021, and initiating professional development and supervision pathways in 2022. These processes will be detailed in the following sections.

## Theoretical underpinning

**Figure 2:**
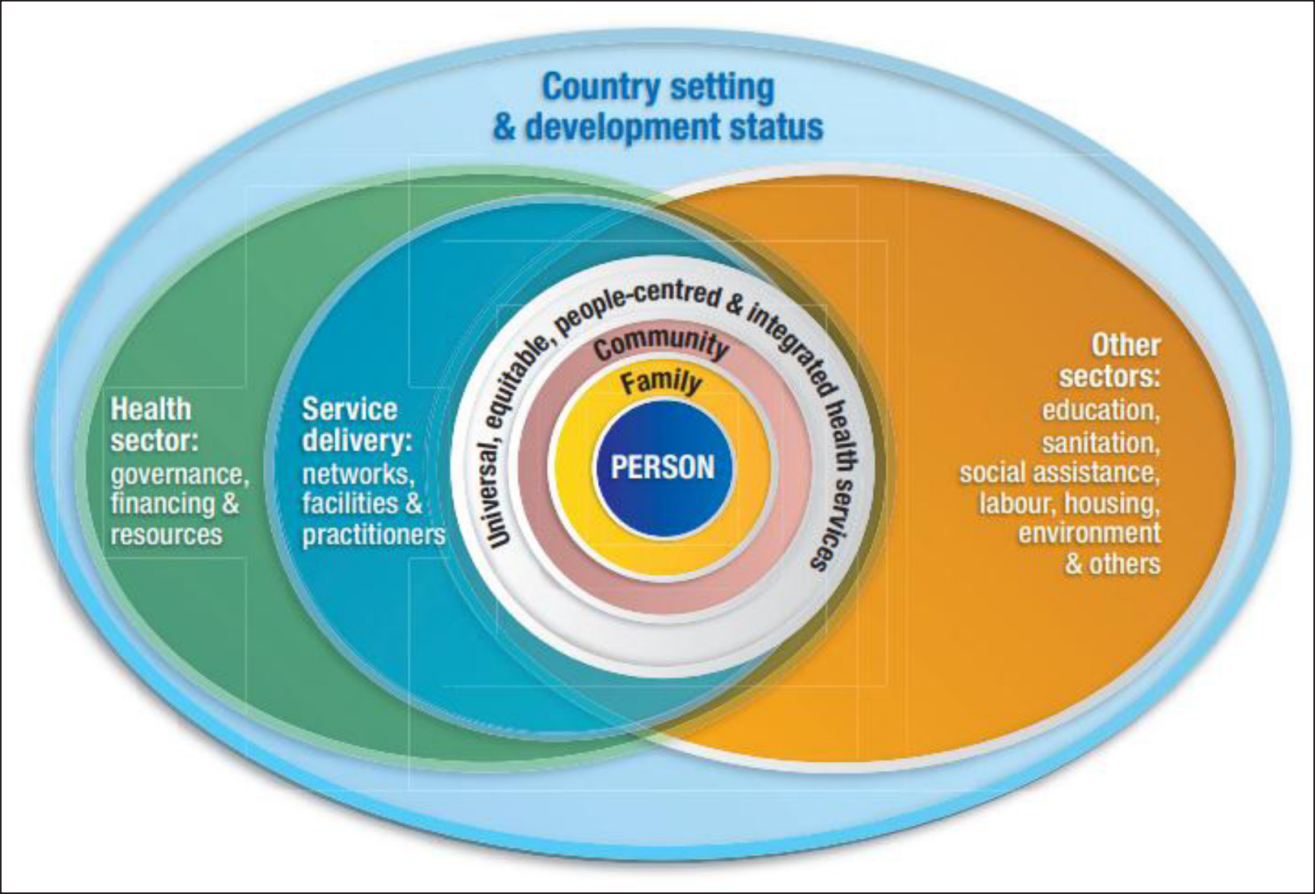
Conceptual framework for integrated people-centred health service [65].

Health and education go hand in hand and are core human rights [64]. The World Health Organisation (WHO) global strategy on integrated people-centred health services (IPCHS) informed the development of NS (Figure 2). The IPCHS strategy encourages a lifespan approach to health, with a shift from curative- or treatment-focused healthcare to health prevention, promotion and protection, particularly in the areas of noncommunicable diseases, mental health and injuries [65]. NS was a person-centred initiative, based on the needs of the community, where health services were re-orientated to be delivered at school to improve access to physical health and mental health care.

The importance of the connection between health and education is emphasised in the WHO policy on ‘Health Promoting Schools (HPS)’ [33]. HPS is a key global standard outlining the potential that embedding healthcare in schools can have across the social determinants of health [33]. Concepts from these standards informed the development of NS, particularly global standard 8 which refers to the comprehensive provision of health and wellbeing services at schools [33].

Locally, the NCMHWS provides an important framework for re-orientating mental health care towards prevention, early intervention, and child-centred practice. The NCMHWS echoes the concepts described by HPS including the importance of equitable access to services and service delivery that utilise integrated family care models [26]. Collaborative and integrated care were an essential element in the design of NS to deliver equitable and accessible healthcare to families with high needs.

When developing NS, cultural knowledge from the local Aboriginal community was drawn upon. For the Aboriginal community, a holistic understanding of the ‘Social and Emotional Wellbeing’ (SEWB) of a child was important. This concept reverberated in the names gifted to the initiative including ‘Ngaramadhi’ which means ‘deep listening’ and ‘Wouwanguul Kanja’ which combines the concept of health and education [12,14,66]. The Aboriginal community voiced their beliefs about the importance of collaborative partnerships between the health and education sectors to understand the ‘whole of the child and family’ which formed the backbone of the NS model of care [67]. These concepts align with the Australian Government’s commitment to drive change in relation to policies and programs affecting Aboriginal people, as highlighted in the Commonwealth ‘Closing The Gap Implementation Plan 2023’. This plan outlines four ‘Priority Reforms’ including formal partnerships and shared decision-making, building the communitycontrolled sector, transforming government organisations, and shared access to data and information at a regional level [68].

The social needs of students were a priority when conceptualising NS. This critical feature was driven by the HHAN framework and aligned with Component 1 of the framework where development of interagency models of care for ‘high need’ schools was prioritised as well as Component 6 which relates to place-based initiatives. Furthermore, the NS model was associated with Component 7 of the HHAN framework because it required system changes and reorientation of healthcare to where it was needed by the community [62,63].

**Figure 3:**
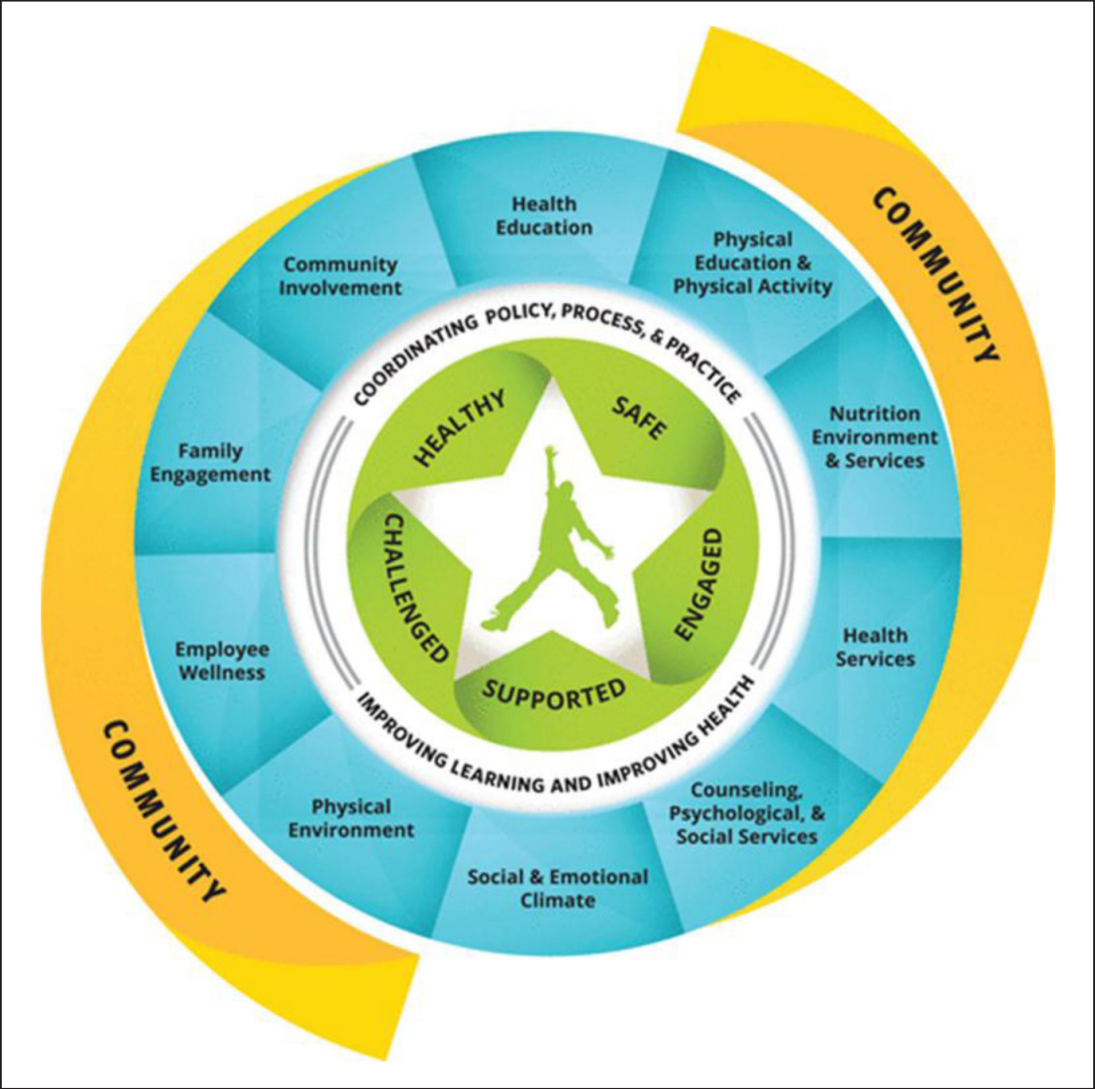
The Whole School, Whole Community, Whole Child Framework (WSCC) [69].

In the U.S, the ‘Whole School, Whole Child, Whole Community (WSCC)’ framework (Figure 3) provides an important guideline for addressing integration of health within schools [69]. While the health and education sectors differ between Australia and the US, the knowledge underlying frameworks such as the WSCC framework remains relevant locally [70,71]. While not prescriptive in nature, the framework describes 10 main components for developing SBHC models [56,70]. The NS model illustrates these key components as well as the underlying concepts of coordinating policy, process and practice (Table 1) [69].

**Table 1:** Examples of how the Ngaramadhi Space model applies to the WSCC framework [69].


WSCC COMPONENT	EXAMPLES FROM NGARAMADHI SPACE

Health education	Provision of education by youth nurses

Nutrition environment and services	Nutritional food provided to students Students involved in food preparation

Employee wellness	Multidisciplinary teams fostering connections Professional development sessions Networking Free staff counselling

Social and emotional school climate	Teachers trained in trauma-informed practice

Physical environment	Purpose-built space

Health services	Multidisciplinary holistic assessments

Counselling, psychological and social services	School counsellor Youthblock services External providers

Community involvement	Wouwanguul Kanja committee

Family engagement	Families attended assessments

Physical education and physical activity	Internal and external providers at school


## Partners and Integrating Care

### Partners and Service Delivery

**Figure 4:**
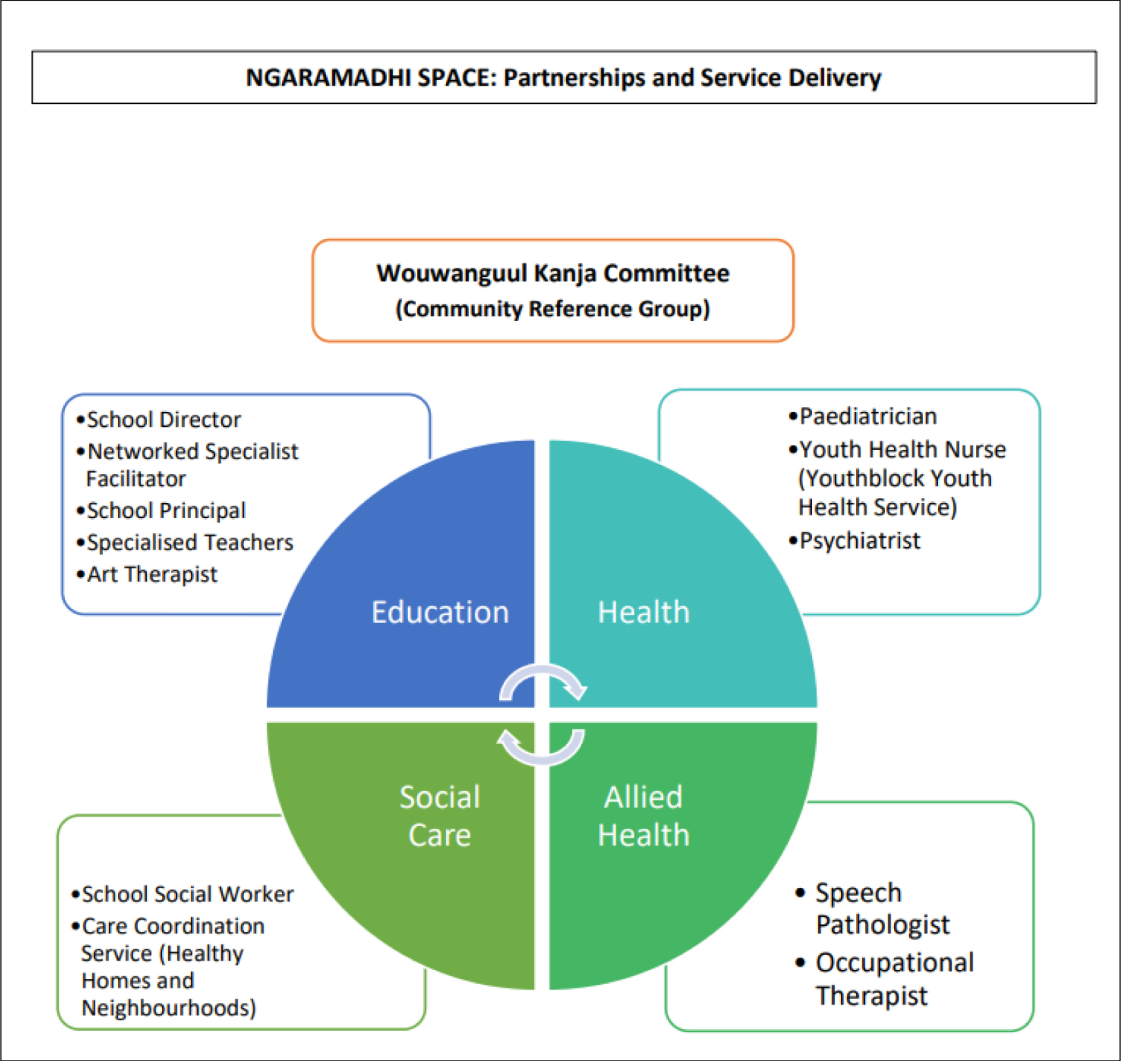
Diagram Illustrating the Partnerships and Service Delivery for Ngaramadhi Space.

NS was built through, and on, partnerships developed over time involving community paediatrics, the school, allied health, and the social care sector, and was overseen by the Wouwanguul Kanja Committee (Figure 4). Key stakeholders from the education sector included the school director, principal, and a networked specialist facilitator, with each holding a unique strategic role. For instance, the school director provided governance and direction to multiple schools within the community and afforded executive level endorsement of the NS model of care, while the principal provided leadership within the school and played an integral role in embedding NS within the school’s existing processes and culture [72,73]. The networked specialist facilitator was a specific position within the Department of Education (DOE), whose role centred on building connections between the education sector and other agencies, including the health sector [74]. Furthermore, the school, due to its specialised nature, provided the model with teachers and therapists skilled in managing behavioural issues.

From the health sector, a community paediatrician, with support from the Director of Community Paediatrics and the Child and Family Health Management team, was integral in driving the formation of the multidisciplinary clinic, providing paediatric developmental and behavioural expertise, and establishing an evaluation process. The Youthblock Youth Health Service (Youthblock) providing the team with a specialist youth health nurse and pathways to support young people e.g. drug and alcohol counselling, sexual and reproductive health services [75]. A child and adolescent from SLHD was partnered with to establish a system whereby the paediatrician could consult with the psychiatrist for advice and support regarding students [76].

The NS model required the skills of a social worker. Initially a non-governmental organisation called Family Referral Service (FRS), now called ‘Family Connect and Support’, was partnered with to assist families in connecting with local support services [77]. Later, the school contracted a social worker for this role. Senior clinicians from the HHAN team had a broad understanding and knowledge of the community and provided support to the NS team [78].

Students at the school were identified as requiring trauma-informed speech pathology (SP) and occupational therapy (OT). The school contracted these therapists using a combination of public and school funding.

### Wouwanguul Kanja Committee

Since its conceptualisation in 2015/2016, community members and stakeholders played an active role in informing the NS model. After the piloting phase (2016–2019), a community reference group called the Wouwanguul Kanja Committee was formally established. The Aboriginal community members decided on the structure of the committee agreeing that it was to be kept small and include representatives from the Aboriginal community and NS team. The Aboriginal members provided insight into culturally safe ways of working including a ‘whole of child and family’ approach and having services co-located and integrated at the school [67]. The community members communicated the importance of the different sectors working together by not only providing a multidisciplinary assessment but also collaborating after the assessment to ensure that outstanding matters could be discussed.

All members of the committee were actively involved in designing the evaluation process, ethics applications, reporting of results and preparing scholarly articles. The committee met regularly and developed agreed upon terms of reference. The community members expressed a need to scale up the NS model because the holistic approach was seen as an effective way of improving access and engagement with health and education services [67,79].

## Model of care

### Referrals and Pre-Assessment Information Gathering

YGS is a specialised behavioural school and acceptance is based on a referrals process. Enrolment at the school and participation in NS were voluntary. During the six-week school orientation period, verbal and written information about the NS assessment was provided. Written permission was obtained from parents to attend the assessment and for information sharing across the sectors. To enable provision of family-centred care, when concerns were raised about siblings of students, they were also offered a NS assessment.

**Figure 5:**
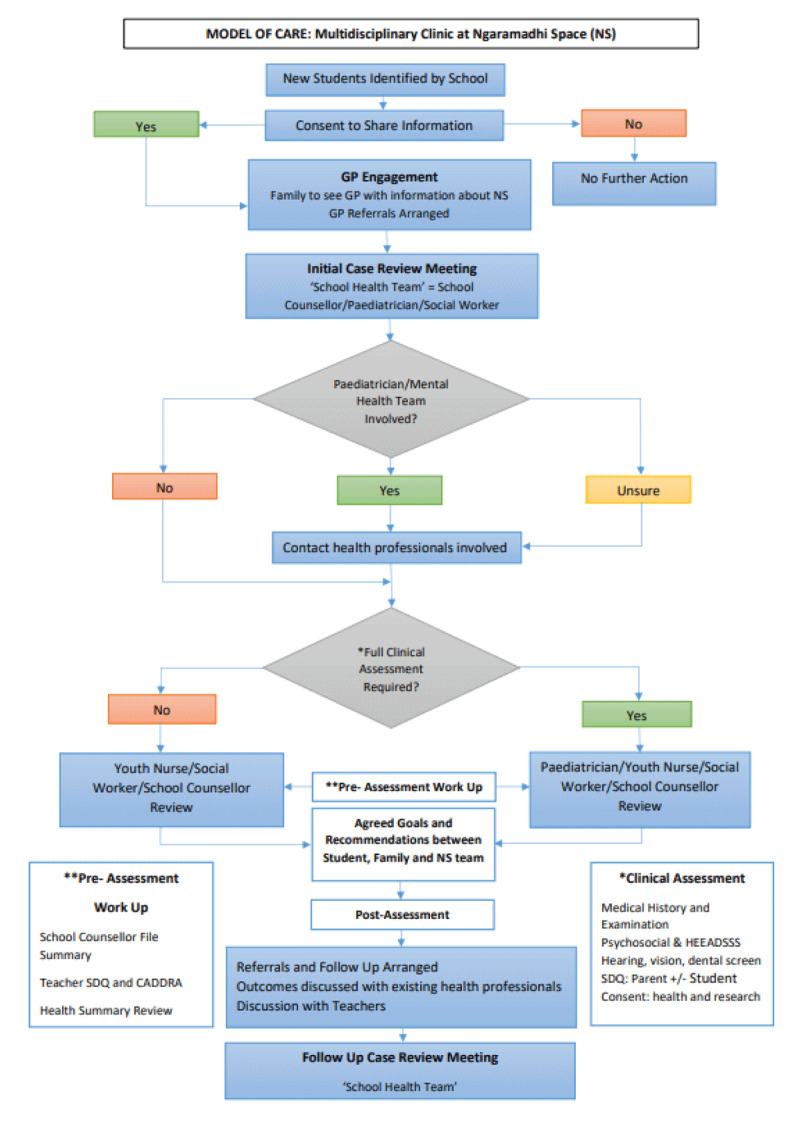
Model of Care for Ngaramadhi Space Multidisciplinary Clinic.

Following the pilot phase of the NS assessment, a consistent approach to the pre-assessment phase was established (Figure 5). A ‘School Health Team’ was developed and included the paediatrician, youth health nurse, social worker, and school counsellor. An initial ‘Case Review Meeting’ was held to discuss referrals and to facilitate information sharing. If a student was engaged with an external paediatrician or psychiatrist this clinician was considered to be the lead clinician. Before proceeding with a full NS assessment, the lead clinician was contacted to see if the assessment would be beneficial. If the lead clinician felt that a full assessment was appropriate, then this would be arranged, and the lead clinician was offered an opportunity to be present for the assessment either in person or using videoconferencing. If the lead clinician did not think a full assessment was necessary, a partial team assessment with a social worker, school counsellor and youth health nurse would be offered to the family. A partial team assessment involved a current psychosocial history without a full medical review.

Collating and sharing information prior to the NS assessment was a key component of the model. Information sharing amongst agencies is necessary when providing high-quality health and social care and the team were guided by NSW privacy-related legislation [80]. The school counsellor summarised information about the family, assessment results, and education support. The class teacher provided current information about the student’s academic skills, behaviour and SEWB by completing a Canadian ADHD Resource Alliance (CADDRA) report and a Strengths and Difficulties Questionnaire (SDQ teacher version) [81,82]. The paediatrician provided a summary of available health information. This preparation led to a better understanding of the student and how to approach the assessment.

Maintaining a ‘medical home’ with the family general practitioner (GP) was an important consideration in developing the model of care [83]. Parents were asked to share written information about NS with their GP to help build this connection. Families also required GP referrals for the clinic and to see the SP and OT. A letter communicating the assessment findings of the NS assessment was provided to the GP as well.

### Multidisciplinary Assessment

The full multidisciplinary assessment was conducted with the student and parent by the School Health Team (Figure 5). After introductions, consent and confidentiality were verbally established. The team approached the student’s health and psychosocial history flexibly, taking into account the student’s behavioural needs and the family dynamics. During the assessment the student was seen on their own by the youth health nurse or paediatrician to conduct an adolescent psychosocial assessment (HEEADSSS assessment), hearing, vision and dental screening, and complete a physical examination including anthropometric measurements [84]. A parent and student SDQ were also completed.

The team then discussed the presenting issues and formed a joint and holistic understanding of the student in line with the family’s priorities. Where possible, students were linked into existing services such as established community paediatric clinics or mental health services. When this was not possible, other avenues within the community were explored. Towards the end of the assessment consent for the separate processes of sharing medical information and participation in the research component was discussed and completed. After the assessment a comprehensive letter was compiled primarily by the paediatrician with input from other team members. Box 1 illustrates a typical patient journey through NS.

**Box 1 Patient journey through Ngaramadhi Space.**

- CJ: 14-year-old Aboriginal male referred to Yudi Gunyi School (YGS) for significant behavioural issues
- YGS orientation: caregiver provided with information about Ngaramadhi Space (NS). Written permission to attend NS and for information sharing was obtained.
- Family visited General Practitioner (GP):discussed NS assessment; referrals for paediatrician, occupational therapist and speech therapist provided.
- School Health Team: collated information including school counsellor summary, medical records and teacher reports (CADDRA and SDQ).
- Initial Case Review Meeting: CJ’s health, education and social information discussed. Issues raised:
  - In kinship foster care since 6 months old
  - Severe meningococcal C meningitis aged 9 months
  - Complex psychosocial trauma.
  - Oppositional defiant disorder (ODD), post-traumatic stress disorder (PTSD), attention-deficit and hyperactivity disorder (ADHD) and depression
  - Intermittent engagement with mental health services
- Mental health team contacted: agreed that a full NS team assessment would be beneficial and to being contacted after the assessment.
- NS assessment: CJ and carer seen by full NS team (Paediatrician, Youth Health Nurse, Social Worker, School Counsellor).
  - Consent and confidentiality discussed
  - Comprehensive medical, education and social history obtained.
  - CJ seen by the youth health nurse: completed HEEADSSS psychosocial assessment; dental, vision and hearing screening.
  - Paediatrician conducted physical examination including growth measurements.
  - School counsellor and social worker completed a parent and student SDQ.
- Multidisciplinary team discussion and findings:
  - Parental substance abuse and domestic violence.
  - Paternal incarceration.
  - CJ attended multiple schools with poor academic achievement
  - CJ’s behaviour was impulsive and aggressive.
  - Police involvement.
  - CJ had recently started self-harming.
  - CJ was the victim of bullying.
  - CJ was using alcohol and vapes.
  - Foster placement threatened because CJ’s behaviour was having a negative impact on carer’s son’s mental health.
  - Physical health issues: possible obstructive sleep apnoea (OSA) and teeth grinding.
- Recommendations
  - School counsellor: emotional regulation strategies
  - Speech pathology and occupational therapy.
  - Social worker: linked CJ with youth police liaison officer provide positive life experiences e.g. sports, mentor.
  - Youth health nurse: drug, vaping and alcohol counselling
  - Counselling arranged for carer’s son
  - Paediatrician: discussed medication options; referred to Ear, Nose and Throat specialist and dentist.
  - Written medical and research consent received.
- Assessment letters distributed to GP and mental health team.
- CJ discussed at staff meeting with psychiatrist in attendance.
- School Health Team met at subsequent Case Discussion Meeting to review CJ’s progress.

### After the Assessment

Students were discussed at the subsequent Case Review Meeting to consider any outstanding issues. Once in every school term the NS team met with school staff to facilitate communication and to understand the student’s progress. The psychiatrist attended these meetings either in person or using videoconferencing to provide insight into further diagnostic or management options.

### Employee Wellness

Working in psychological trauma services can have an impact on staff wellbeing [70]. Participating in a multidisciplinary model helped alleviate individual burden of care. In addition, regular professional development sessions and support networks were established to promote connectedness across the sectors. Staff were also aware of how to access free employee counselling.

### Evaluation Methods and Initial Outcomes

NS is a complex intervention where students have diverse needs and attend a multidisciplinary assessment [85]. NS is being evaluated using a mixed methods design and these results will be reported in later manuscripts using the model of care described in this paper. Preliminary quantitative evaluation has shown that students had multiple unmet physical health, mental health, and social needs. Attendance at the NS was high and there was a statistically significant improvement in teacher reported scores using the SDQ [in print]. A qualitative study examining how the education and health sector can work in partnership including facilitating factors and barriers is currently underway and will be reported in a later manuscript. The authors acknowledge that because this was a pilot study, data on longitudinal outcomes were not evaluated. The research team are designing an implementation study with the following outcome measures:

**Access:** Proportion that access care prior and after SBHC implemented, number of service encounters and referrals, outcomes of referrals

**Health outcomes:** Prior and new diagnoses

**Socio-Emotional Wellbeing:** SDQ at initial visit, 6 months and 12 months.

**Education outcomes:** School attendance before and after SBHC, suspensions, referrals and supports.

**Implementation outcomes:** Reach, adoption of service integration of services, implementation in different contexts, implementation barriers and facilitators, acceptability and appropriateness

**Economic evaluation**

## Discussion

The NCMHWS states that ‘in an optimal [health and] mental health system, all doors lead to help and services are designed and delivered based on the needs of children and families’ [26]. As we head into a post-pandemic era we are confronted with a widening of pre-existing health and socioeconomic disparities [21,24,25]. Novel approaches to ‘building it back differently’ are needed with the unmet needs of young people high on the global agenda [23,30].

The NS model of care builds on national and international strategies to integrate care. The following section will discuss how effective the model of care was and which areas could be improved through the lens of the IPCHS framework [65].

### Engaging and empowering people and communities

The NS model of care was effective in engaging and empowering people and communities. This was achieved through effective community consultation which led to the development of a culturally-safe and multidisciplinary model. This approach aligns with the Closing The Gap Implementation strategy which emphasises the role of formal partnerships and shared decision-making with the Aboriginal community [68].

YGS is unique and has an important role in the community, acting as an endpoint for students experiencing significant behavioural issues. The trajectory for these students is poor and the community saw a need to change this pathway. A holistic approach was considered important in addressing the multifactorial issues that resulted in the problematic behaviour. For the Aboriginal community it was critical that the SEWB of students was recognised, a term which reflects the important connection between physical, mental and emotional health with social, spiritual and cultural wellbeing [12,14,18]. Amongst the Aboriginal community SBHC is an effective way to address inequity with collaborative partnerships between the health and education sector that seek to understand the ‘whole of the child and family’ valued [67,79]. NS was governed by a community reference group, Wouwanguul Kanja, that oversaw all aspects of the design and evaluation of the initiative. This active participation from the community led to acceptance of the model along with calls to scale up the model so that other students could benefit from the approach [67,79]. It is important for this level of community participation to be maintained and improved upon as the model evolves and is scaled up.

### Coordinating services within and across sectors

The NS model achieved coordination within and across sectors through sharing of information, joint assessments and case review meetings. Working across sectors allowed timely and effective management of learning and behavioural difficulties. For example, health staff could directly contact teachers about the effectiveness of medication changes. This level of communication meant that the likelihood of complex students slipping through the system was reduced and allowed staff to present consistent messages to students and families [53].

Collaboration across sectors often presents challenges. Time is required for teams to understand each other’s role, work through conflict, develop trust and effective communication [86,87]. Some issues that arose included an understanding of who the ‘leader’ was and assigning responsibility for tasks [88,89,90]. There were matters arising from changes in staffing and changes in stakeholder capacity as well. The impact of these incidents was minimised by taking a stepwise approach to implementing the model and documenting roles and responsibilities [89].

### Strengthening governance and accountability

Over many years the NS model was able to strengthen governance and accountability across sectors. The health and education systems are complex and hierachical systems. Navigating these systems and bringing them into alignment required persistence, relationship development and time with decision-making by School Health Teams often requiring endorsement at a high executive level [53].

The direction provided by the Wouwanguul Kanja community group helped embed NS within the school. Through this avenue a MOU was agreed upon. In the context of NS, creating a MOU helped legitimise the way the various sectors worked together in view of setting a precedent for other models when scaling up the initiative. Further to these processes, administrative and educational meetings were set up for the whole team and supervision was strengthened by linking the school social worker with the HHAN care coordination team. These are small steps towards true integration and further high-level executive support is required within the departments of health and education.

### Reorienting the model of care

Health and education are closely linked across the lifespan. Healthy students are in a better position to learn and students with higher education levels are more likely to engage with healthy behaviours throughout their life [11,91,92,93]. The NS model is a school-based, holistic, and integrated care initiative. By reorientating health service delivery to schools access to care is improved thereby providing an opportunity to intervene and interrupt the trajectory of those attending YGS.

Delivering health services at schools is convenient for students and families while allowing health staff to leverage of the trust students have with the school. This approach has been shown to improve engagement, particularly for priority populations such as ethnic minority groups and those from low socioeconomic areas [34,43,47].

### Creating an enabling environment

For the previous four strategies to become operational, it is important to create an enabling environment. NS was embedded within the overall HHAN framework [62,63]. This strategy helped bring together key stakeholders to undertake transformational change [65]. This was a complex task involving changes to the way staff from the various sectors worked together. The school principal played an important role in establishing a welcoming environment for the different sectors to connect and in embedding the NS processes within the school’s workflow and culture. The NS team needed to learn how to adapt to changing circumstances, pivoting to meet the needs of the school and community, particularly during the COVID19 pandemic. The multidisciplinary team shared a common purpose and demonstrated a willingness to work flexibly and come to the school to improve access and engagement of students. The team continued to revise and refine the model of care, a process that occurred over years so that person-centred care could be optimised. The team developed a MOU to provide structure to how the sectors worked together including how the model of care could be sustained. An evaluation process was embedded in the model so that the quality of service delivery could be improved upon, a process which continues to be developed for future studies. NS is a pilot model of care and to ensure that it can be scaled up further commitment at a state-level from health and education is required.

## Conclusion

Health and education are key social determinants of wellbeing [30,43,94]. The high disease burden associated with developmental and behavioural concerns in children, further potentiated by the COVID19 pandemic, has created an urgency for collaborative responses that can be translated into policy and be scaled up [22,24,25]. By incorporating health care into education settings children and their families are given access to excellent integrated care [53].

In Australia, there is a need for innovation that is culturally responsive and where community members have a real and tangible role in the co-design of services [12,18,26,79]. Schools are an under-utilised community space within Australia and the health sector can play a pivotal role in improving the wellbeing of children by forming effective partnerships with schools [26,52]. NS is an example of a model that brings together all these elements and provides an opportunity within the Australian context to address inequitable access to health and mental health care. The model of care has been developed over a decade and has been informed by the community and stakeholders. The Aboriginal community have voiced the need for similar models of care in addressing access issues for Aboriginal families as well as all Australians. The community acceptance of this type of model provides an important first step in addressing inequitable access and can form a base for prevention and early intervention models [26]. Furthermore, the NS model of care is centred on understanding the student in the context of ‘people, place and land’ thereby recognising the role of culture, society and history on behaviour [79].

## Key Points

- Ngaramadhi Space (NS) is a novel and integrated child and family school-based healthcare initiative for students enrolled at a specialised school for problematic externalising behaviour.
- The NS model of care is community-led and accepted as a way to reduce health and education inequities.
- SBHC is a re-emerging area within Australia with a growing interest in scaling up such initiatives.
